# Bleeding Esophageal Varices and Portal Hypertension Caused by Arteriovenous Fistula of Splenic Artery

**DOI:** 10.1155/1990/49734

**Published:** 1990

**Authors:** Moshe Shleapnik, Baruch Shpitz, Annette Siegal, Alex Dinbar

**Affiliations:** 1 Department of Surgery ‘B’ Meir General Hospital Kfar Saba and the Tel Aviv University Sackler Faculty of Medicine Tel Aviv Israel; 2 Department of Pathology Meir General Hospital Kfar Saba and the Tel Aviv University Sackler Faculty of Medicine Tel Aviv Israel; 3 Head, Department of Surgery ‘B’ Meir General Hospital Kfar Saba 44 281 Israel

## Abstract

Splenic arteriovenous fistula is a rare but curable cause of portal hypertension. This report describes a patient with such a disorder, presenting with bleeding esophageal varices and ascites. It emphasises the importance of performing selective catheterization of the celiac and superior mesenteric artery in all patients with signs of portal hypertension without evidence of chronic liver disease. Etiopathology and management are discussed.


*HPB Surgery*, 1990, Vol. 3, pp. 53–57
Reprints available directly from the publisher
Photocopying permitted by license only

© 1990 Harwood Academic Publishers GmbH
Printed in the United Kingdom

### BLEEDING ESOPHAGEAL VARICES AND PORTAL HYPERTENSION CAUSED BY ARTERIOVENOUS FISTULA OF SPLENIC ARTERY

MOSHE SHLEAPNIK, BARUCH SHPITZ, ANNETTE SIEGAL* and ALEX DINBAR

*Departments of Surgery 'B' and *Pathology, Meir General Hospital, Kfar Saba, and the Tel Aviv University Sackler Faculty of Medicine, Tel Aviv, Israel.*

*(Received 7 February 1990)*

Splenic arteriovenous fistula is a rare but curable cause of portal hypertension. This report describes a patient with such a disorder, presenting with bleeding esophageal varices and ascites. It emphasises the importance of performing selective catheterization of the celiac and superior mesenteric artery in all patients with signs of portal hypertension without evidence of chronic liver disease. Etiopathology and management are discussed.

KEY WORDS: Bleeding esophageal varices, portal hypertension, arteriovenous fistula, splenic artery

### INTRODUCTION

Splenic arteriovenous (A-V) fistula is a rare cause of portal hypertension. It presents mostly as upper gastrointestinal bleeding due to esophageal or gastric varices with or without ascites[1–5]. Early diagnostic celiac trunk arteriography is mandatory in patients with presenting symptoms suggestive of portal hypertension, but without evidence of liver disease. The relative ease and the success of sclerotherapy, which has progressively supplanted surgical shunting, may create a tendency not to seek an underlying cause for bleeding varices and an otherwise curable form of portal hypertension may be overlooked.

### CASE REPORT

A 64-year-old multiparous woman was admitted to the internal medicine department because of diffuse abdominal pain and distension of several weeks duration. She complained of weakness and weight loss. There was no history of trauma, previous surgery, hepatitis or alcoholism. Abdominal examination revealed ascites and painless splenomegaly. The liver was not enlarged and there was no jaundice.

Address correspondence to: Prof. A. Dinbar, Head, Department of Surgery 'B', Meir General Hospital, Kfar Saba 44 281, Israel

All the laboratory data, including the liver function and coagulation tests were within normal limits. Alfafetoprotein and Australian antigen were negative. Abdominal ultrasound and CT scan showed ascites, splenomegaly and significantly enlarged splenic and portal veins. A barium swallow demonstrated esophageal varices. A percutaneous liver biopsy obtained tiny liver fragments; only one small portal space could be seen with mild fibrosis and few lymphocytes. Ascitic fluid cytology was negative for malignancy.

In spite of a non-conclusive liver biopsy, the patient was discharged under the assumed diagnosis of chronic liver disease. During the following two months she was admitted twice because of hematemesis, treated by blood transfusions, vasopressin and endoscopic sclerotherapy. She was lastly admitted to the surgical department with massive hematemesis and signs of hypovolemic shock (blood pressure 70/50 mmHg and pulse rate 120/min). Laboratory data revealed 17,000 WBC/cc, Hb 9.9 gldl, Na 120 mEq/1, K 4.8 mEq/1, PT 70%. After rapid fluid and blood resuscitation followed by a vasopressin drip (0.8 units/min for 12 hours, then decreased to 0.4 units/min for two days), a Sengstaken-Blakemore tube was inserted. The bleeding stopped and 24 hours later endoscopic sclerotherapy was performed. On the following day the patient had a new episode of bleeding. An emergency celiac trunk arteriography was done (Figures 1 and 2), demonstrating a large A-V fistula between the splenic artery and vein, with early filling of a markedly dilated portal vein, splenomegaly and distended veins communicating with the portal system.

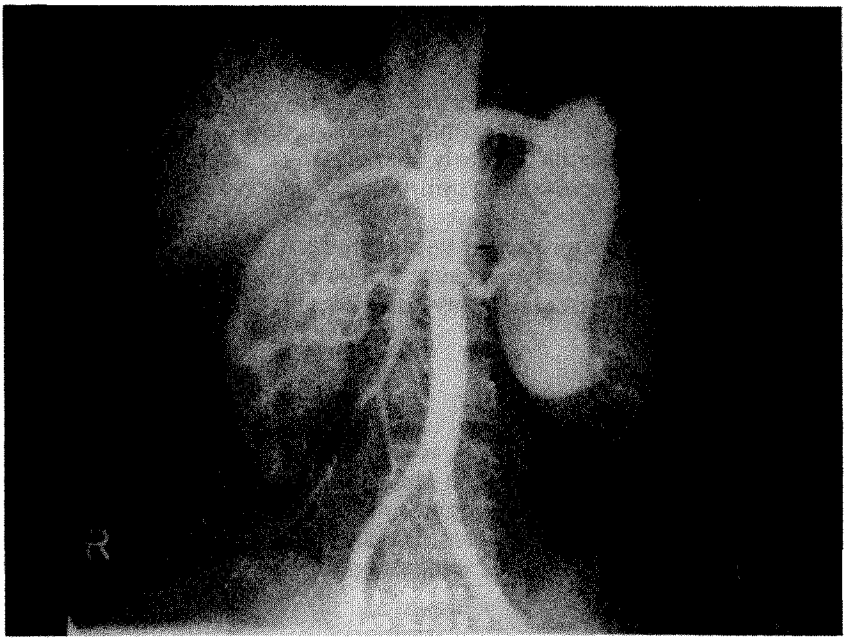

**Figure 1.** Celiac trunk arteriography demonstrating: A. A large A-V fistula between the splenic artery and vein.

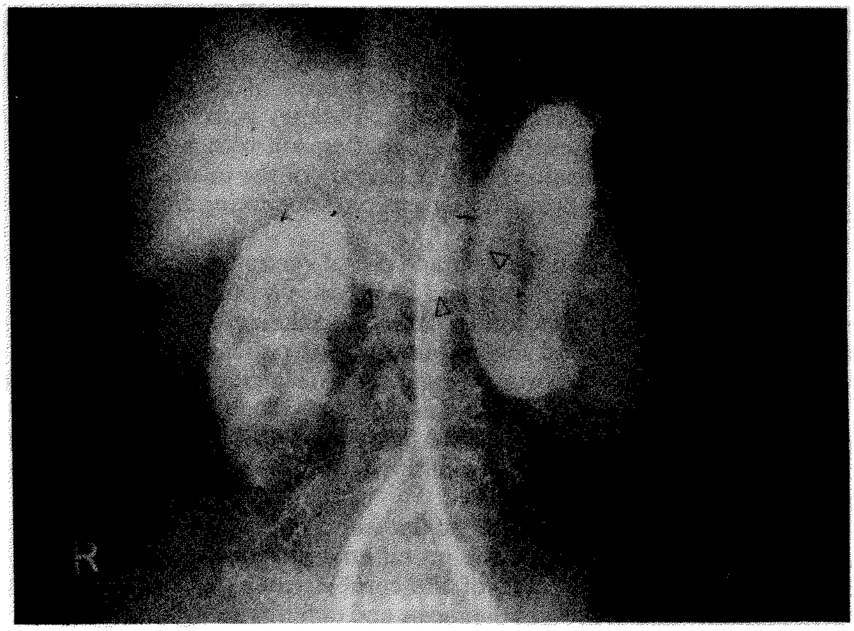

**Figure 2.** Early filling of a dilated portal vein.

At laparotomy, approximately 6 liters of ascitic fluid were removed. A large splenic artery and vein were visualized with fistula between them, located near the splenic hilus. Severely distended veins were found along the lesser and greater curvatures of the stomach and in the retroperitoneum lateral to the tail of the pancreas and in the greater omentum. The spleen was enlarged. The fistula was resected and splenectomy performed. Multiple varices were ligated along the lesser and greater curvatures of the stomach. She was discharged five weeks later after surgical drainage of a left subphrenic abscess.

At six months follow-up, the patient remained asymptomatic and in excellent condition. Laboratory data, including the liver function tests, are normal.

*Pathology*: The spleen weighed 850 g and revealed several large acute infarcts and moderate interstitial fibrosis. In the hilar fat, at the site of the fistula, the venous segment showed a thickened muscle and intimal fibrosis with focal mucoid changes. Small and fresh thrombi were attached to the endothelium. The arterial segment showed artherosclerosis with focal calcifications. Adjacent to a small pancreatic tissue fragment removed together with omental fat were seen tortuous and dilated veins with thickened walls and a few mural thrombi attached. A liver tissue fragment from underneath the capsule showed dilated sinusoids, mild portal fibrosis with congested, mildly thickened, portal venous radicals.

### DISCUSSION

Splenic A-V fistula, first described in 1886, is a rare condition which can present with symptoms related to portal venous hypertension and congestion, such as abdominal pain, dyspepsia, ascites and splenomegaly. Repeated episodes of upper gastrointestinal bleeding due to esophageal or gastric varices are also frequent[1,2]. They are thought to arise in part from erosion of acquired[6] or congenital[7] splenic artery aneurysm into the splenic vein. Other causes of fistulization are trauma,[5] postsplenectomy[8] or rare mycotic infections[3]. Ascites is infrequently seen and is attributed to long standing severe portal hypertension with systematization of the portal venous circulation, resulting in hypertrophy of the intrahepatic portal veins. These changes intensify the portal hypertension, accounting from accumulation of ascitic fluid[4].

Morphological alterations in the liver and portal venous system in systemic portal arteriovenous fistula have been described in few case reports and studied mainly in the experimental animal[9]. The term "hepatoportal sclerosis" was used and the histological changes resembled those found in our patient, i.e. fibrosis of portal triads and arteriolization of portal circulation. Arida[4] reported a case of splenic A-V fistula with esophageal varices and ascites without any histological changes of the intrahepatic vascular bed. The fresh infarcts in the large spleen in our case could be due to embolization of the multiple mural thrombi found in the venous part of the fistula.

Selective catheterization of the celiac and superior mesenteric arteries remains the method of choice for imaging the portal anatomy whenever an A-V fistula is suspected. Since this lesion is one of the curable causes of portal hypertension, early arteriography allows prompt diagnosis and definitive surgical treatment of this rare disease. Selective embolization has been proposed and successfully performed by a few authors[10]; however, splenectomy with fistulectomy and variceal ligations seems to be the treatment of choice whenever portal hypertension is complicated by gastrointestinal bleeding, severe varicosities and ascites.

### *References*

1. Curran, D.H., McCaughan, G., Waugh, R. and Stephen, M. (1984) Curative treatment of bleeding esophageal varices secondary to a splenic arterio-venous fistula. *Australia and New Zealand Journal of Medicine* **14**, 849–851.
2. Pang, D. (1985) Arterioportal fistula as cause of hypertension. *Mt. Sinai Journal of Medicine* **51**, 644–646.
3. Van Way, C.W. III, Crane, J.M., Riddell, D.H. and Foster J.H. (1971) Arteriovenous fistula in the portal circulation. *Surgery* **70**, 876–890.
4. Arida, E.J. (1977) Splenic arteriovenous fistula with portal hypertension, varices, and ascites. *New York State Journal of Medicine* **77**, 987–990.
5. Gudmendsen, T.E., Lie, M. and Ostensen, H. (1988) Splenic arteriovenous fistula. *Acta Chirurgica Scandinavica* **154**, 603–604.
6. Harlan Stone, H., Jordàn, W.D., Acker, J.J. and Martin, J.D. (1965) Portal arteriovenous fistulas — Review and case report. *American Journal of Surgery* **109**, 191–196.
7. Bradfeldt, J.E. and O'Laughlin, J.C. (1980) Portal hypertension secondary to a congenital splenic arteriovenous fistula. *Journal of Clinical Gastroenterology* **2**, 355–356.
8. Bartside, R. and Famelli, R.L. (1987) Splenic arteriovenous fistula. *Journal of Trauma* **27**, 671–673.
9. Donovan, A.J., Reynolds, T.B., Mikkelsen, W.P. and Peters, R.L. (1969) Systemic portal arteriovenous fistula: pathological and hemodynamic observations in two patients. *Surgery* **66**, 470–482.
10. Keller, R.S., Rosch, J. and Dotter, C.T. (1980) Bleeding from esophageal varices exacerbated by splenic arterio-venous fistula: complete transcatheter obliterative therapy. *Cardiovascular Intervention and Radiology* **3**, 97–102.

*(Accepted by S. Bengmark 7 February 1990)*

### INVITED COMMENTARY

This is an interesting single case report, as the authors say, of a "rare but curable cause of portal hypertension". It serves however to underline strongly the need for full investigation of patients with portal hypertension in order to identify patients such as this who have a completely curable cause. Furthermore it highlights the fact that each patient needs to have his or her treatment tailored to the underlying pathology.

It also reinforces the desirability of all patients with portal hypertension being referred to a centre which possesses the appropriate expertise in all diagnostic and therapeutic modalities. Without such across the board experience patients will inevitably be inappropriately treated on occasions. In this case, because of the diagnostic skill and dexterity of the surgical team, a very satisfactory outcome ultimately resulted. The more conventional sclerotherapy, which many believe to be the "treatment of choice", failed.

K.E.F. Hobbs
University of London
London, U.K.

